# Primary leiomyosarcoma of epididymis: a case report

**DOI:** 10.1186/s13256-024-04660-7

**Published:** 2024-07-21

**Authors:** Hong-Jie Chen, Yao-dong Han, Dong-hai Li, Wu Li, Jun Zhang

**Affiliations:** 1 Department of Urology, The First Ren Ming Hospital of Lanzhou, Lanzhou, China; 2 Department of Pathology, The First Ren Ming Hospital of Lanzhou, Lanzhou, China; 3 Department of Imageology, The First Ren Ming Hospital of Lanzhou, Lanzhou, China

**Keywords:** Leiomyosarcoma, Epididymal, Sarcoma

## Abstract

**Background:**

Leiomyosarcoma is a tumor that can develop in any organ that contains smooth muscles. Although leiomyosarcoma is common, its epididymal localization is quite rare.

**Case presentation:**

A 79-year-old male Chinese Han patient presented with mild pain in the right groin and scrotum for 3 years concomitant with right scrotal swelling. Ultrasonography and magnetic resonance imaging of the scrotum showed a irregular and heterogeneous mass that was extratesticular. Right high orchiectomy was performed, and pathological examination of the resected specimen confirmed the diagnosis of leiomyosarcoma of the epididymis with surgical margins clear of tumor.

**Conclusion:**

Epididymal leiomyosarcoma is rare and difficult to diagnose preoperatively. The final diagnosis of epididymal leiomyosarcoma requires histologic examination. Resection must be extensive and complete. The effect of chemotherapy and radiation on the epididymal leiomyosarcoma remains unclear. Recurrence is common, so follow-up is necessary.

## Background

Leiomyosarcoma (LMS) is a malignant mesenchymal tumor arising from the smooth muscle, the vascular smooth muscle, or the mucous muscle of the intestinal wall, accounting for 5–10% of all soft tissue tumors [1]. The LMS of peritesticular tissue was derived from the testicular tunica (48%), spermatic cord (48%), epididymis (2%), and dartos muscle and scrotal subcutaneous tissue (2%) [2]. Epididymal LMS is rare and occurs in the smooth muscle surrounding the basement membrane of the epididymal duct [3]. Due to its rarity, there is no sufficient evidence regarding the ideal workout for diagnosis, treatment, and follow-up.

## Case presentation

**Fig. 1:**
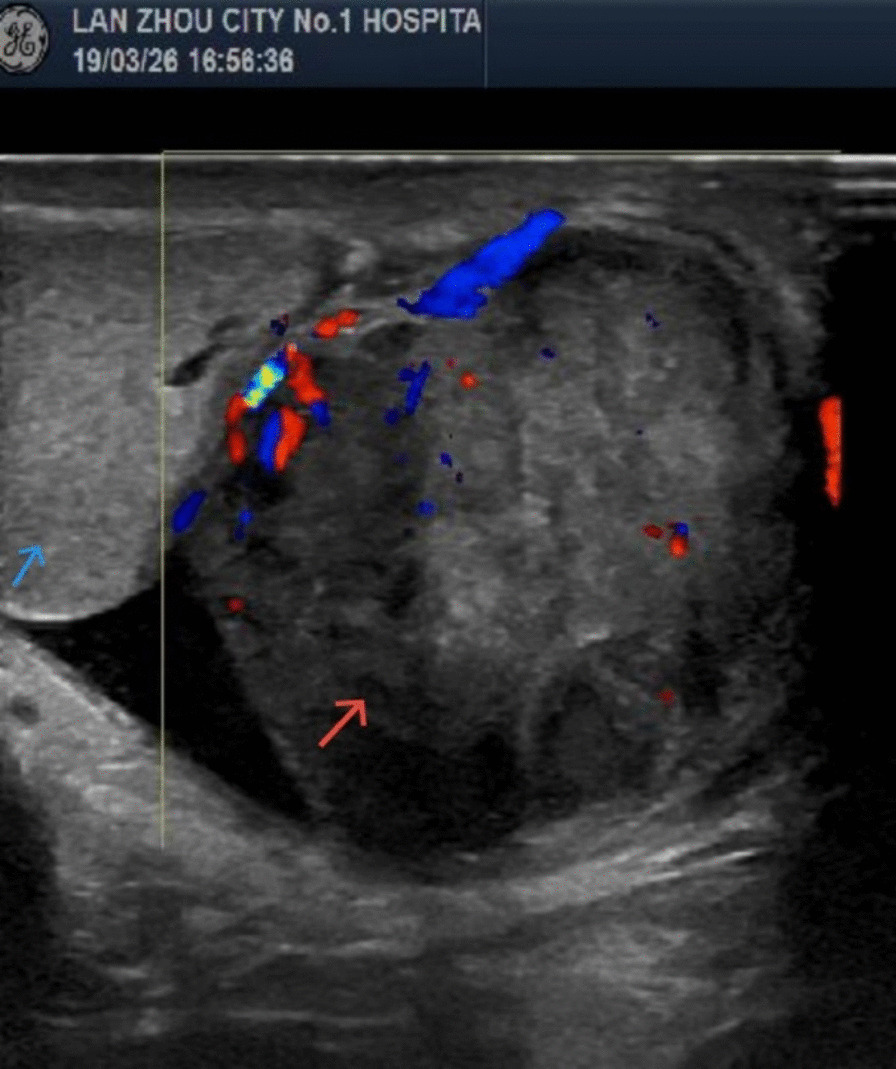
Scrotal ultrasound showing a well-defined hypoechoic paratesticular mass (red arrow) located in the inferior aspect of the right testicle (blue arrow), measuring approximately 4.5 cm × 3.5 cm × 4 cm

**Fig. 2:**
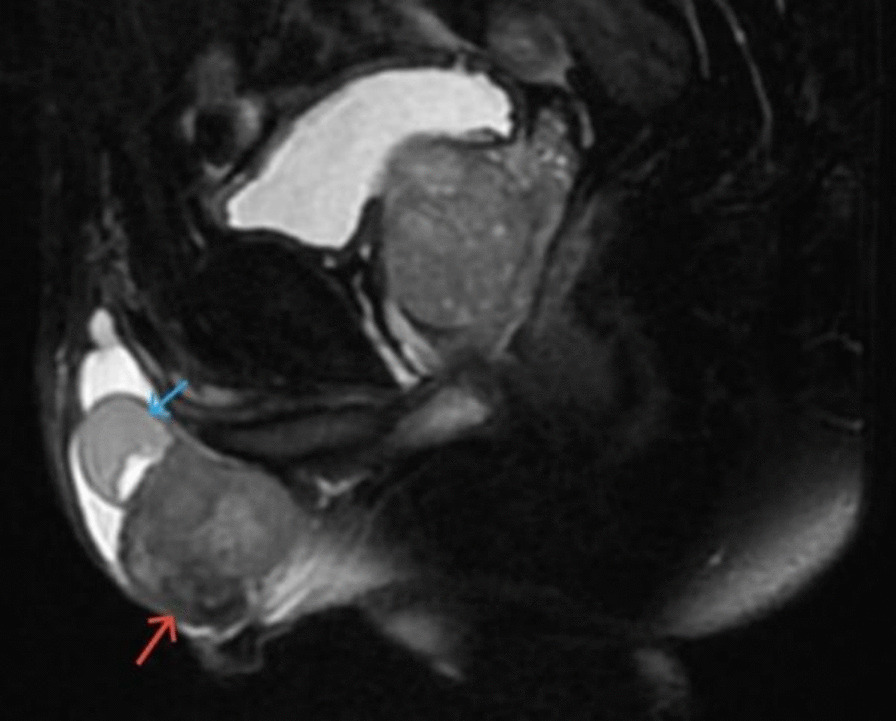
Magnetic resonance imaging showing that the mass (red arrow) around the right testicle (blue arrow) was about 3.5 cm × 3.5 cm × 4.0 cm in size and oval in shape

A 79-year-old male Chinese Han patient presented to his urologist with mild pain in the right groin and scrotum for 3 years concomitant with right scrotal swelling. There was no context of a significant social, family, or personal experience occurring before or at the onset of the symptoms and no history of trauma, urinary tract infection, hematuria, dysuria, or surgery. Physical examination revealed a 4.5 cm × 3.0 cm × 4.0 cm swelling arising from the lower pole of the right testicle. The mass was painless, palpable, and hard. Scrotal ultrasound identified a well-defined hypoechoic paratesticular mass located in the inferior aspect of the right testicle, measuring approximately 4.5 cm × 3.5 cm × 4.0 cm (Fig. 1). Magnetic resonance imaging (MRI) showed that the mass around the right testicle was about 3.5 cm × 3.5 cm × 4.0 cm in size and oval in shape (Fig. 2).

**Fig. 3:**
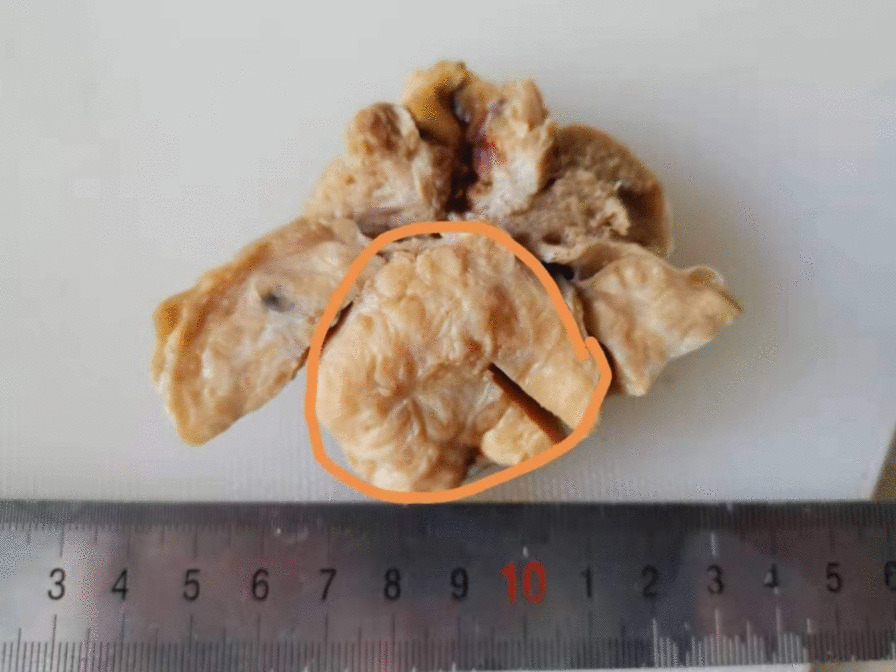
Gross specimen revealing a 4 cm × 3.5 cm × 3.5 cm solid tumor mass whose cut surface was grayish white with a crisp texture (orange circles)

**Fig. 4:**
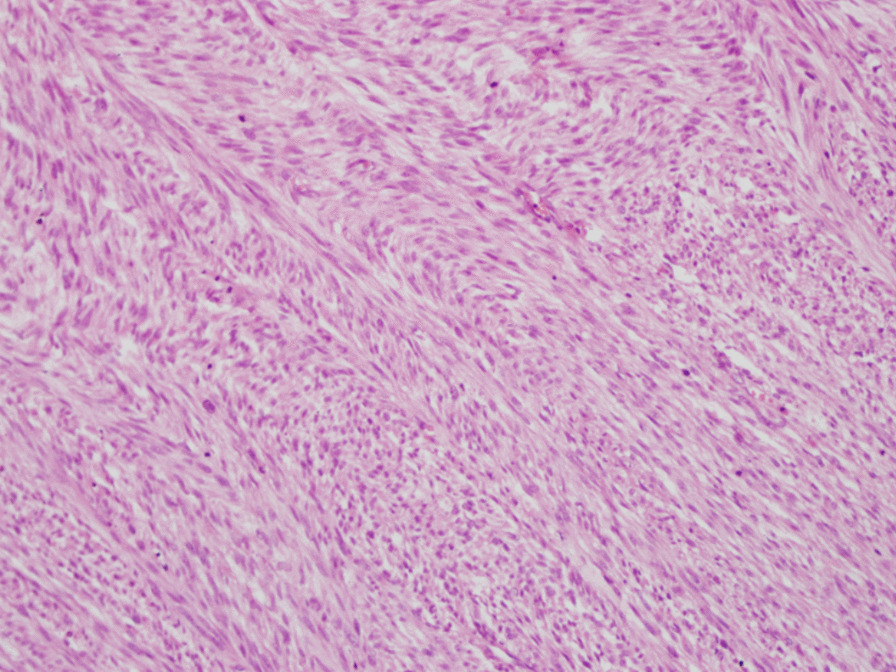
The tumor was composed of pleomorphic spindle cells arranged in fascicles (hematoxylin-eosin staining ×200)

**Fig. 5:**
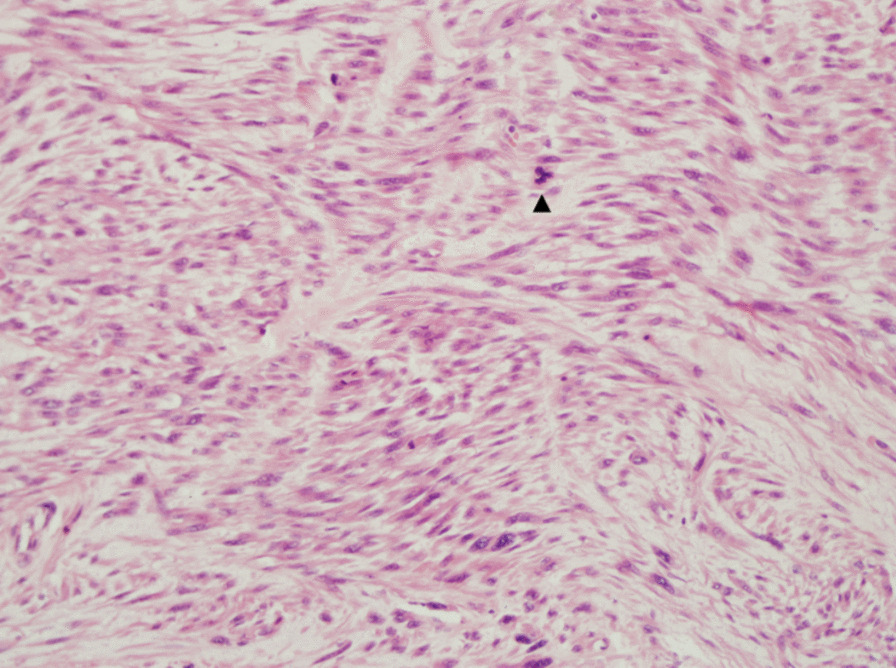
The tumor cells were markedly heterogeneous, with pathological mitosis, as shown by the black arrow (hematoxylin-eosin staining ×400)

**Fig. 6:**
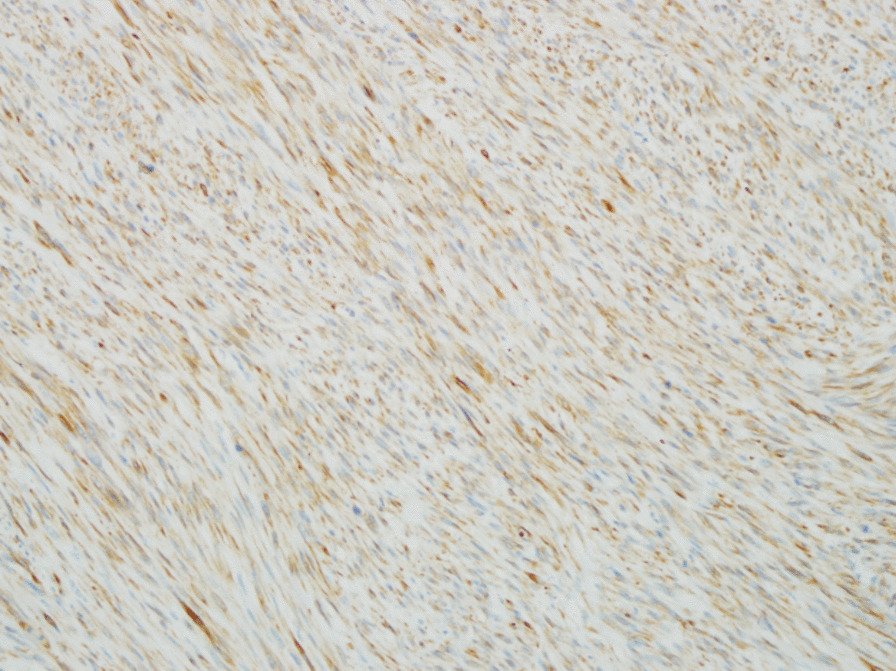
Desmin positive (immunohistochemistry ×200)

**Fig. 7:**
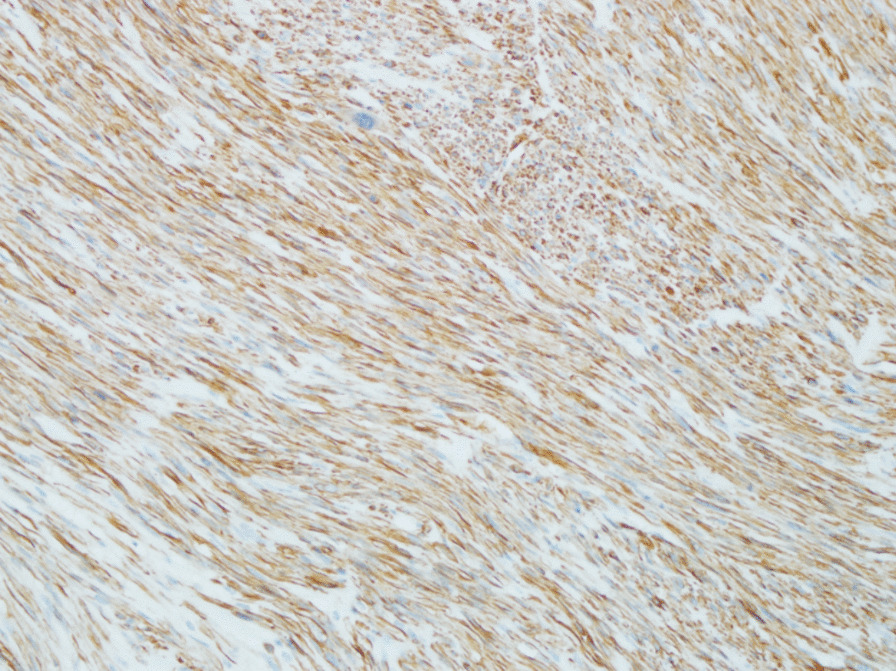
H-caldesmon positive (immunohistochemistry ×400)

Right high orchiectomy was performed. The macroscopic view was reported as a lobulated 4.0 cm × 3.5 cm × 3.5 cm solid tumor mass whose cut surface was grayish white with a crisp texture (Fig. 3). On histopathology, the tumor was composed of pleomorphic spindle cells arranged in fascicles (Fig. 4) and the tumor cells were markedly heterogeneous, with pathological mitosis (Fig. 5), invading the albuginea testis, and grade 1 (according to National Federation of French Cancer Centers and National Cancer Institute system). Immunohistochemistry showed tumor cells to be positive for smooth muscle action (SMA), desmin (Des), h-caldesmon, vimentin, and Epithelial membrane antigen (Figs. 6, 7) and negative for CD34, CD117, PLAP, a-inhibin, ki-67, DOG-1, myogenin, MyoD, S100, and SOX10. The pathological diagnosis was primary epididymal LMS. After the operation, chest and abdominal computed tomography (CT) scans were performed, and tumor markers were detected. No abnormalities were found. He was not planned for any adjuvant therapy. The patient is still being followed up regularly.

## Discussion

Kwae *et al.* [4] in 1949 claimed the first case report of primary epididymal LMS. Epididymal LMS is more common in men aged 50–80 years, but it may also occur in children and young adults [5]. Risk factors for paratesticular LMS include high doses of anabolic steroids, chronic inflammation, or past exposure to radiation [6], but there are no reported predisposing factors leading to epididymal LMS in the literature. Epididymal LMS is difficult to diagnose preoperatively, and the typical clinical presentation is of a painless, firm, slow-growing, intrascrotal mass, with palpation usually revealing the mass to be well defined, lobulated, mobile, and sometimes associated with a small hydrocele. The examination should begin with an ultrasound of the scrotum to determine the size and location, texture, and vascular distribution of the mass. Song *et al.* [7] describe the sonographic features of epididymal LMS: Epididymal LMS demonstrates sonographic characteristics common to many malignant tumors, such as increased density, irregular shape, heterogeneous internal echogenicity, and hypervascularity. Ultrasonography is helpful to differentiate benign epididymal lesions and can provide some reference for clinical diagnosis and treatment. However, compared with other malignant tumors of the epididymis, it has no characteristic sonographic features. MRI may be better at locating the tumor and elucidating its relationship to surrounding tissue in more detail [8].

Histologic examination of a surgically resected specimen and morphological and immunohistochemical evaluation is needed for definitive diagnosis. The classic histologic features are rhomboid, fasciculate, and braided arrangement of tumor cells, marked cell atypia, and obvious mitosis [9]. Immunohistochemistry: SMA(+), desmin(+), S-100(−), CD34(−), CD117(−) [10].

Current consensus is to perform radical orchiectomy with high ligation of the spermatic cord, when a diagnosis of epididymal malignant tumor is made [11]. Kamitani *et al.* [12] performed a retrospective analysis of 217 reported cases of paratesticular LMS. Patients treated by simple tumorectomy were reported to have a significantly higher risk of a positive surgical margin (9 of 17 versus 5 of 27, *p* = 0.024), which they described to be an independent risk factor for local recurrence. However, there was no significant difference in terms of distant metastasis (DM) and disease-specific survival (DSS) between simple tumorectomy and high inguinal orchiectomy. But Tchienga *et al.* [13] suggest that low-grade and localized tumors with negative margins can be managed with simple epididymectomy and imaging surveillance. The effect of adjuvant therapy (chemotherapy and radiation) on the epididymal LMS remains unclear, and there is a need for further investigation [14]. Dehghani *et al.* [15] recommend adjuvant therapy if histopathology is diagnosed with LMS.

Locoregional recurrence patterns that are reported in the literature include the following: scrotal [16], inguinal and retroperitoneal [17], and even gastrointestinal mucosal, extremities, and lung metastases [18, 19]. The experiences above, and other documents and literature, strongly support the importance of long-term follow-up for all the patients.

## Conclusion

Epididymal LMS is rare and difficult to diagnose preoperatively. The final diagnosis of epididymal LMS requires histologic examination. Resection must be extensive and complete. The effect of chemotherapy and radiation on the epididymal LMS remains unclear. Recurrence is common, so follow-up is necessary. Because most cases reported in the literature are retrospective analyses of case reports, small series, literature reviews, and expert opinions, they show different outcomes depending on several variables. In future work, we still need to summarize a large number of cases to further confirm the diagnosis and treatment of epididymal LMS. The purpose of this article is to delineate the clinicopathologic features of epididymal LMS and spread awareness of the malignant nature of the disease, to improve the diagnosis and treatment of this disease.

## Data Availability

Not applicable.
